# Female Genital Mutilation/Cutting Resulting in Genital Tract Obstruction and Sexual Dysfunction: A Case Report and Literature Review

**DOI:** 10.1155/2021/9986542

**Published:** 2021-08-10

**Authors:** Anwar Sadat Seidu, Haruna Danamiji Osman, Kingsley Appiah Bimpong, Kwame Afriyie

**Affiliations:** ^1^Department of Surgery, Tamale Teaching Hospital, Box TL 16 Tamale, Ghana; ^2^Department of Surgery, School of Medicine, University for Development Studies, Box TL 1350 Tamale, Ghana; ^3^Department of Obstetrics and Gynecology, Greenshield Hospital, Box 193, Sefwi Bekwai, Ghana

## Abstract

Female Genital Mutilation/Cutting (FGM/C) is the practice of cutting parts of the female external genitalia in fulfillment of sociocultural obligations and in some cases for nonmedical reasons. It is classified into 4 main types depending on the extent of cutting. Some forms of FGM/C are common in at least 29 countries globally, mainly in Africa. The overall prevalence of FGM/C in Ghana is approximately 4%. The motivation for this practice varies from community to community but includes the fulfillment of cultural values, uplifting the girl child, and, according to some reports, reducing sexual desire and promiscuity. The objective of this article is to illustrate how FGM/C resulted in sexual dysfunction in a young woman married for 2 years. We present a 19-year-old female who was subjected to female genital cutting in her formative years who presented with apareunia for 2 years in her marriage. We illustrated how FGM/C led to a genital tract obstruction with resultant sexual dysfunction. Examination revealed a Type 3 FGM/C (infibulation) with almost complete occlusion of the genital tract. She underwent a successful defibulation and resumed sexual activity with her husband within 6 weeks of the procedure.

## 1. Introduction

Female Genital Mutilation (FGM) is the term used by the World Health Organization (WHO) to refer to all medically unnecessary cutting of the external female genitalia, often carried out to fulfill perceived sociocultural obligations [1, 2]. This term is increasingly criticized by scholars for being applied selectively to non-Western-associated forms of female genital cutting (FGC) and for conflating multiple distinct practices with different risk profiles [3]; thus, we will use the term Female Genital Mutilation/Cutting (FGM/C) to acknowledge this debate. This compromise is increasingly used by scholar-clinicians [4]. The WHO distinguishes 4 main types of FGM/C depending on the extent of cutting: Type 1: partial or complete excision of the external, visible portion of the clitoris, primarily the glans (clitoridectomy); Type 2: partial or complete excision of the external clitoris and labia minora (excision); Type 3: narrowing of the genital tract opening from cutting and repositioning the labia (infibulation); and Type 4: other medically unnecessary skin-breaking procedures affecting the female genitalia such as piercing, pricking, or nicking [5].

Yoder et al. reported that over 100 million women have been affected by FGM/C globally and a further 3.3 million girls are affected yearly [6]. Some forms of FGM/C are common in at least 29 countries globally, mainly in Africa, with a reported prevalence between 0.6% and 97.9% in Uganda and Somalia, respectively [2]. In recent years, scholars have also highlighted practices of FGM/C in Southeast Asia, for example, in Malaysia and Indonesia [7]. If one does not count Western-associated so-called “cosmetic” female genital cutting, which some scholars argue technically meets the WHO definition of FGM/C [8], there are only a small number of reported cases in Europe and the United States, primarily among immigrants [6]. The prevalence is over 90% in Somalia, Guinea, Djibouti, and Egypt. In Ghana, the prevalence is 4% with about 1.3 million women affected by the practice thus far [1]. It is especially common in the northern part of Ghana where the prevalence is about 38% [9]. The most common form of FGM/C in this area, namely, excision [10, 11], is associated with documented complications such as bleeding, pain, wound infections, sexual dysfunction, obstetric complications, and even death [1, 2, 12, 13].

## 2. Case Presentation

A 19-year-old circumcised girl from Northern Ghana presented to the hospital with complaints of apareunia since she got married 2 years before presentation. She had no lower abdominal pain, vaginal discharge, or fever. She had no past medical or surgical history. She had her menarche at 14 years and had regular monthly cycles, with 7-day flow, and no dysmenorrhea or menorrhagia. She had not had any formal education. She and her partner are farmers.

**Figure 1:**
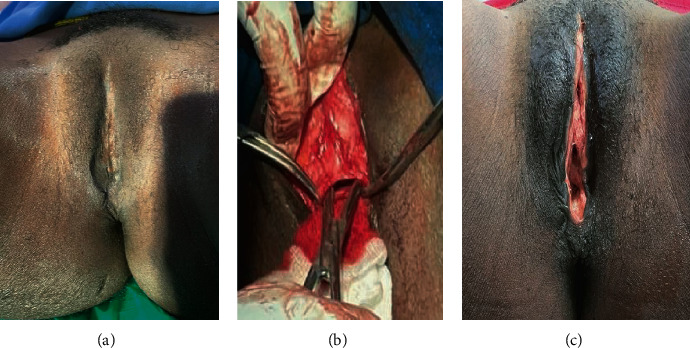
(a) Vulva with excised clitoris and labia majora and minora with obliteration of the vaginal opening, (b) defibulation, and (c) one month postop.

On examination, she was stable, not pale, and looked well nourished. Normal blood pressure readings were obtained. Her breast was well developed. The abdomen was flat, soft, and nontender with no masses palpated. Examination of the vulva revealed scarred external genitalia with dry skin. The glans clitoris, prepuce, right and left frenula, and the labia minora and majora were absent as shown in Figure 1(a). There was a pinhole vaginal orifice amid the scarred tissue. Complete blood count (CBC) and Renal Function Test (RFT) were essentially normal. Abdominopelvic ultrasonography showed a normal anteverted uterus, no myomas, normal endometrial stripe, no adnexal mass, and no free fluid in the pouch of Douglas.

She was counseled for defibulation, to be performed under spinal anesthesia. A stab incision was made at the lower margin of the vulva and a Kelly clamp inserted under the scar tissue and a vertical incision made along the anterior surface of the infibulated scar until the original anatomical site of the clitoris was exposed superiorly and introitus inferiorly as shown in Figure 1(b). The clitoris was not buried under the scar tissue. The cut edges were separated with a gauze dressing and povidone ointment. The vagina and cervix were inspected, but no pathology was found. The urethral orifice was seen in its normal position. She was discharged the next day and scheduled for follow-up visits. Wounds healed without complications as shown in Figure 1(c). Sexual intercourse with her husband was initiated within 6 weeks of the procedure without difficulty.

## 3. Discussion

FGM/C prevalence in Ghana varies by region with a relatively high prevalence around 38% in Northern Ghana [9, 12, 14]. In this report, the patient was a young female who came from the northern part of Ghana but migrated to settle in the southern part of Ghana. She was subjected to this act in her formative years in the rural part of Northern Ghana. An early study by Sakeah et al. noted that religion and ethnicity were key factors for this practice and that males from Northern Ghana with little or no education are more likely to prefer circumcised women [15], but a recent study has shown that men's preferences do not drive the practice in Northern Ghana [16].

In Africa, countries like Ghana and Senegal took initiatives by way of legislation to criminalize FGM/C. In 1994, an Act was enacted to amend the criminal code of Ghana, making FGM/C a criminal offense equivalent to second-degree felony liable to imprisonment of at least 3 years [17]. However, this piece of legislation has had little effect on the practice of FGM/C because of noncollaboration between opinion leaders, state institutions, and human rights groups [18].

In the northern part of Ghana, it has been noted that FGM/C is common among daughters of women who have little or no form of education [14]. Akweongo et al. also highlighted the pivotal roles of parents in FGM/C as mothers have been seen to be the encouragement behind daughters getting circumcised with fathers providing the permission [16].

The patient in this report is a teenager who was subjected to infibulation as a minor as part of a sociocultural practice. She has lived with the consequences of this practice since it happened until the present moment. FGM/C is still being practiced in Ghana [14]. Berg and Denison in their systemic review in 2011 reported that women who undergo at least some forms of FGM/C are more likely to experience dyspareunia and reduced sexual satisfaction [19]. Apareunia which was reported in our case was also reported in an earlier case report in a 31-year-old woman who suffered FGM at 10 years [20]. Good sexual response encompasses multiple factors including psychosocial and good sexual stimulation. Berg and Denison noted that women who have had FGM/C were more likely to have reduced sexual desire and dyspareunia [19]. The scarred tissue could result in stenosis and in some instances complete obstruction of the genital tract as seen in this case report. Okwudili and Chukwudi reported a case of a 23-year-old girl who presented with both urinary and genital tract obstruction in Nigeria [13]. Documented complications of FGM/C include prolonged labor, perineal tears, increased likelihood of cesarean sections, stillbirth, postpartum hemorrhage, chronic perineal pain, dyspareunia, sexual dysfunction, and obstetric fistula [21–23].

The pressing health problems of the patient we saw seem to have been resolved. However, a long-term follow-up would be needed to determine whether she may have other consequences, including potential psychological concerns [23].

## 4. Conclusion

We reported a case of a patient experiencing sexual difficulties due to infibulation. Defibulation allowed this patient to engage in sexual intercourse with her partner within 6 weeks following surgery.

## Abbreviations

FGM: Female Genital Mutilation
FGM/C: Female Genital Mutilation/Cutting
WHO: World Health Organization.
